# Human Metapneumovirus Establishes Persistent Infection in Lung Microvascular Endothelial Cells and Primes a Th2-Skewed Immune Response

**DOI:** 10.3390/microorganisms8060824

**Published:** 2020-05-30

**Authors:** Antonella Bugatti, Stefania Marsico, Manuela Fogli, Sara Roversi, Serena Messali, Daniela Bosisio, Cinzia Giagulli, Arnaldo Caruso, Silvano Sozzani, Simona Fiorentini, Francesca Caccuri

**Affiliations:** 1 Section of Microbiology, Department of Molecular and Translational Medicine, University of Brescia, 25123 Brescia, Italy; antonella.bugatti@unibs.it (A.B.); manuela.fogli@yahoo.it (M.F.); s.roversi003@unibs.it (S.R.); s.messali@unibs.it (S.M.); cinzia.giagulli@unibs.it (C.G.); arnaldo.caruso@unibs.it (A.C.); francesca.caccuri@unibs.it (F.C.); 2 Department of Pharmacy, Health and Nutritional Sciences, University of Calabria, Arcavacata di Rende, 87036 Cosenza, Italy; stefania.marsico@unical.it; 3 Section of General Pathology and Immunology, Department of Molecular and Translational Medicine, University of Brescia, 25123 Brescia, Italy; daniela.bosisio@unibs.it (D.B.); silvano.sozzani@unibs.it (S.S.)

**Keywords:** *human metapneumovirus*, dendritic cells, Th2 response, IL-4, OX40L

## Abstract

*Human Metapneumovirus* (HMPV) is a major cause of lower respiratory tract infections. HMPV infection has been hypothesized to alter dendritic cell (DC) immune response; however, many questions regarding HMPV pathogenesis within the infected lung remain unanswered. Here, we show that HMPV productively infects human lung microvascular endothelial cells (L-HMVECs). The release of infectious virus occurs for up to more than 30 days of culture without producing overt cytopathic effects and medium derived from persistently HMPV-infected L-HMVECs (secretome) induced monocyte-derived DCs to prime naïve CD4 T-cells toward a Th2 phenotype. Moreover, we demonstrated that infected secretomes trigger DCs to up-regulate OX40L expression and OX40L neutralization abolished the pro-Th2 effect that is induced by HMPV-secretome. We clarified secretome from HMPV by size exclusion and ultracentrifugation with the aim to characterize the role of viral particles in the observed pro-Th2 effect. In both cases, the percentage of IL-4-producing cells and expression of OX40L returned at basal levels. Finally, we showed that HMPV, *per se*, could reproduce the ability of secretome to prime pro-Th2 DCs. These results suggest that HMPV, persistently released by L-HMVECs, might take part in the development of a skewed, pro-Th2 lung microenvironment.

## 1. Introduction

Virus infections are the primary cause of respiratory illness in humans and, in particular, RNA viruses, such as *respiratory syncytial virus* (RSV), *human metapneumovirus* (HMPV), parainfluenza, and influenza viruses, are the most common pathogens that are associated with lower respiratory tract infections. HMPV causes a spectrum of respiratory illness, ranging from mild upper respiratory tract infections to severe bronchiolitis and pneumonia [1], clinically indistinguishable from that caused by the related RSV [2,3,4,5]. Serological studies suggest that HMPV, which has a worldwide distribution, is acquired early in life and, by age of five years, approximately 70% of all children develop antibodies to HMPV [6].

Primary cellular targets of HMPV infection actively contribute to the initiation of the host immune response, defining functional properties of tissue-residing dendritic cells (DCs) and, consequently, educating the outcome of adaptive immunity [7,8,9].

Early HMPV infection during infancy is one of the most significant independent risk factors for the development of pre-school asthma [10]. Nasal washes from infants with upper or lower respiratory tract HMPV infection contain increased amount of interleukin (IL)-4 and lower levels of proinflammatory cytokines and IL-12 when compared to secretions that were obtained by children infected with other respiratory viruses [11,12]. Accordingly, the stimulation of peripheral blood mononuclear cells (PBMCs) obtained from experienced healthy subjects with HMPV induces a higher level of IL-6 (a cytokine that prevents Th1 differentiation) and a low level of interferon (IFN)-γ when compared to RSV [13,14]. In vivo, HMPV infection is associated with long-term pulmonary inflammation, which leads to significant obstructive disease of the airways [15,16]. Taken together, these evidences suggest that HMPV might favor a polarization toward a Th2 phenotype. A more recent study has demonstrated that HMPV regulates a complex mixed Th1/Th2 immune [17], where the predominance of a Th2 phenotype might contribute to incompletely eliminating infected cells within the airways following primary infection. Moreover, studies that were performed in immunized mice have shown that primary HMPV infection elicits weak, innate, and aberrant adaptive immune responses that are characterized by the induction of Th2-type cytokines, particularly at later stages of infection. Interestingly, this event coincides with virus persistence in the lung [8,17]. HMPV persistence has been studied in depth while using permissive small animal models. Virus can be recovered months after the beginning of infection, despite the presence of efficient humoral immunity [15,18,19,20]. Further, it has been shown that a cellular reservoir, like neuronal cell, can sustain a viral burst following immunosuppressive treatment [20]. On the other side, HMPV persistence in the human host has been hypothesized, but, so far, little is known regarding its possible cellular reservoirs. Recently, we have shown that HMPV infection is maintained for as long as more than two years in a subset of epithelial cells by overcoming apoptosis. These cells may act as a reservoir of infectious virus that could contribute to in vivo viral persistence [21].

The microvascular endothelial cells (ECs) of the pulmonary capillaries are in intimate contact with the lung epithelial cells. Arnold and König [22] showed that, in the case of RSV infection of the lower respiratory tract, endothelial cells may be exposed to a relevant dose of infectious viral particles and may represent a cellular target of infection. Moreover, growing evidences have shown that orchestration of the innate immune response to respiratory viruses derives not only from lung epithelial cells, but also from ECs [22,23]. ECs are common target of viral infections, which are capable of sustaining acute, as well as persistent, infection in the absence of obvious cytopathic effects [24,25,26]. This peculiar feature suggests that ECs can represent a privileged site of viral persistence in the body.

In this paper, we explored the possibility that lung endothelium might represent a site of HMPV infection and persistence. Further, we aimed to investigate whether EC-derived HMPV was able to trigger a pro-Th2 immune response through its interaction with DCs.

## 2. Materials and Methods

### 2.1. HMPV Production and Titration

HMPV (NL-001 strain, kindly provided by Dr. Osterhaus) was propagated in the Rhesus monkey kidney cell line LLC-MK2 (Istituto Zooprofilattico Sperimentale, Brescia, Italy). LLC-MK2 cells were grown in T-175 flasks in Minimum Essential Medium (Sigma Aldrich, Milan, Italy) that was supplemented with 10% foetal bovine serum (FBS), 0.2% glucose, 1% bovine serum albumine, and 50 μg/mL gentamycin until they were 70% confluent. Cell infection was performed in OptiMEM (Invitrogen, Milan, Italy) in the presence of 1 μg/mL of trypsin and absence of serum. The cells were inoculated with 0.5 multiplicities of infections (MOI) of HMPV in 10 mL of medium and, after three hours (h) of adsorption, an additional 10 mL of medium was added to the flasks. When cytopathic effects first appeared, usually after 5–7 days of culture, supernatant was collected and stored at −80 °C until use. The flask was subjected to two freeze-thaw cycles for 10 minutes (min.) and cell lysate was clarified and purified by sucrose gradient, as described [27]. Virus was then aliquoted and stored at −80 °C until use. A single inoculum was prepared and used for all infection experiments. The inactivation of aliquots of viral stock was achieved by UV treatment for 1 h (1.2 J/cm^2^). To check viral inactivation, UV-treated HMPV was used to infect LLC-MK2 cells. The absence of viral replication was assessed by monitoring the lack of expression of viral antigens by flow cytometry and analysing viral replication by specific real-time (rt) PCR. Mock-infected cell cultures were obtained from uninfected LLC-MK2 cells, processed exactly as the HMPV-infected ones.

Virus titration was performed by limit dilution on monolayer of LLC-MK2 cells that were cultured for 14 days. The first dilution without cytopathic effects was considered the infecting load. Virus titer was also confirmed by plaque titration assay. LLC-MK2 were seeded (2.5 × 10^4^ cells per well) in 96-well plates in culture medium. The following day the infection was performed while using four-fold dilutions of produced HMPV in OptiMEM (final volume 20 μL). After incubation (3 h at 37 °C), the cell monolayer was washed twice with phosphate buffered saline (PBS) and 50 μL of a 0.8% solution of Methylcellulose (SigmaAldrich, Milan, Italy) was added. After 48 h, cells were washed in PBS, fixed in Acetone-Methanol (60/40 *v*/*v*), and saturated with PBS-Bovine serum albumine (2.5%). Subsequently, the cells were stained with a 1:300 dilution of a primary antibody (Ab) to the HMPV Matrix (M) protein (Mab8510, Chemicon, Temencula, CA, USA) and then incubated 45 min at 37 °C. Upon a second 45 min incubation step with biotinylated Anti-Mouse IgG (1:225) (Vector Laboratories, Burlingame, CA, USA), the substrate Vectastain ABC-AP (Vector Laboratories) was added. Finally, the monolayers were counterstained with a 5% methyl-green solution (in 0.1 M sodium-acetate pH 4.2) and infected cells were enumerated.

HMPV genome equivalents in cell lysates and/or supernatants were quantified, as described [21]. Briefly, viral RNA was extracted using a semiautomatic method (NucliSENS EasyMAG, Biomerieux, Florence, Italy), retrotranscribed, and amplified by rtPCR. In house designed primers/TaqMan probe set and calibration standards were used to calculate HMPV absolute copy number.

### 2.2. Endothelial Cell Culture and Infection

Lung human microvascular endothelial cells (L-HMVECs) were purchased from Clonetics (LONZA, Caravaggio, Italy) and cultured in endothelial basal medium that was supplemented with 5% FCS and growth factors (EGM-MV2 BulletKit; LONZA), as described [28]. All of the infections were performed on second to fifth passage L-HMVECs. Briefly, 5 × 10^5^ cells were seeded cells in collagen-coated T-25 flasks (Biocoat, BD Biosciences, Milan, Italy) and, after 24 h, were washed with PBS and mock-infected or infected with 0.5 MOI of HMPV. After a 3 h absorption at 37 °C, the viral inoculum was removed, cells were washed twice with PBS and then incubated with fresh EC medium. Cells and/or culture supernatants were harvested at specific time points for subsequent analysis. Supernatants collected at different times after infection were filtered through 0.45-µm pore membranes and used to infect target LLC-MK2 cell in order analyse the infectivity of L-HMVECs-released virions, as described above.

### 2.3. Immunofluorescence Analysis

Uninfected or HMPV-infected L-HMVECs, which were grown onto collagenated culture slides (Falcon, Milan, Italy), were fixed with cold methanol/acetone (30/70 *v*/*v*) at different times post-infection (PI). The slides were stained with Ab Mab8510 (1:400) directed to the HMPV matrix protein M, followed by the Alexa Fluor 488-conjugated anti-mouse IgG diluted 1:200 (Invitrogen, Milan, Italy). Fluorescence was detected using a Nikon Eclipse TE2000-S microscope (Nikon, Tokyo, Japan).

### 2.4. DC Generation

Peripheral blood samples were obtained from healthy donors. Informed consent was provided according to the Declaration of Helsinki. The PBMCs were isolated by density gradient centrifugation while using Lymphocyte Separation Media (Biosera, Monza, Italy). The DCs were prepared, as previously described [29], with minor modifications. Briefly, monocyte purification was obtained by positive selection, while using anti-CD14 conjugated magnetic microbeads (Miltenyi Biotec, Bologna, Italy). Cell purity, as assessed by flow cytometry (FACS), was always ≥ 95%. Freshly purified monocytes were cultured for five days at the density of 350,000 cells/ml in complete medium (RPMI 1640, 10% endotoxin-free FBS, 2 mM L-Glutamine, and 1% Penicillin-Streptomycin) supplemented with 20 ng/mL of recombinant human IL-4 and 50 ng/mL of granulocyte-macrophage colony-stimulating factor (GM-CSF) both purchased from Miltenyi Biotec to obtain immature DCs (immDCs).

### 2.5. Coculture and Priming of Naïve T Cells

Naïve CD4 T cells were isolated from Buffy Coat of voluntary donors (Blood Bank, Spedali Civili, Brescia, Italy) using CD4^+^CD45RA^+^ T cell Isolation Kit II (Miltenyi Biotec). Purity ≥ 97% was usually obtained. Naïve T cells were cocultured in complete medium with allogenic DCs (T/DCs ratio: 10/1). After seven days of coculture, the cells were stimulated for 8 h by phorbol-12-myristate-13-acetate (PMA) (10 ng/mL) and Ionomycin (1 μM) (Sigma–Aldrich). Brefeldin A (5 µg/mL) (Imgenex, Milan, Italy) was added the last 3 h of stimulation. FACS analysis of intracellular IFN-γ, IL-4 expression was then performed on CD4^+^ T cells gate. When indicated an anti-human OX40L neutralizing antibody was added to the coculture [30].

### 2.6. Flow Cytometry

The production of cytokines by CD4^+^ T lymphocytes was evaluated by a three-color antibody staining (CD4, IFN-γ, and IL-4). The cells were first incubated 30 min on ice with PerCP-conjugated anti-CD4^+^ antibody (BD Biosciences, Milan, Italy), fixed and permeabilized while using the Cytofix/Cytoperm Kit (BD Biosciences) following the manufacturer suggestions. Intracellular staining for cytokines was then performed by incubating cells 30 min on ice with a biotinylated anti-IFN-γ monoclonal antibody (clone IMGB17, produced in our laboratory). After washing, the cells were incubated with APC-conjugated Streptavidin (Invitrogen) and anti-IL-4 PE-conjugated antibody (clone 8D4-8, Biolegend, London, UK), washed, and analysed by FACS.

DCs were incubated in ice for 30 min with specific APC-conjugated anti-human OX40L (Clone IK-1, BD Pharmingen) in 200 μL of PBS containing 2% FBS, washed, and analysed in order to evaluate the expression of OX40L on cell surface. To set parameters, cells that were stained with isotype-matched irrelevant mAb were always used.

The quantification of HMPV infected cells was evaluated by detecting viral antigens at the intracellular level. To this purpose, cells were harvested, fixed with paraformaldehyde, and stained for 30 min at 4 °C while the anti-M clone 8510 (1:250) followed by Alexa-488-conjugated anti-mouse IgG (Invitrogen, Milan, Italy). The uninfected cells were used as control.

Flow cytometric analyses were performed using a FACSCalibur flow cytometer (BD Biosciences), and a range of 5000–15,000 gated cells was acquired. The data were analysed by CellQuest software (BD Biosciences).

### 2.7. Real-Time PCR

RNA extraction, retro-transcription (RT), and rtPCR were performed, as already described [31]. The expression of OX40L specific mRNAs was evaluated using a specifically designed real time assay (OX40L-Forward: 5′-AGGCCAAGATTCGAGAGG-3′; OX40-Reverse: 5′-CCTTTCTCCTTCTTATATTCGGTAAAT-3′). To calculate differences in OX40L mRNA expressions, β-actin mRNAs were used in each experimental sample as housekeeping reference transcripts. All of the samples were amplified in triplicate. The data obtained were analysed with 2^-ΔΔ*C*t^ method comparing the treatments to the mock condition (calibrator).

### 2.8. Thymic Stromal Lymphopoietin (TSLP) ELISA

Fifty μL of L-HMVEC secretomes derived from three different HMPV infections, collected at different time PI (3, 7, 10, 14, 21 days), were analysed for the contents of human Thymic Stromal Lymphopoietin (TSLP). TSLP ELISA Development Kit (Peprotech, London, UK) was used according to the protocol that was provided by the supplier.

### 2.9. Metagenomic Analysis of HMPV

HMPV viral stock was extracted using RNA RNeasy^®^ Mini kit (QIAGEN) following the Manufacturer protocol, and then libraries for metagenomic analysis were generated using the Sequence-independent Single-Primer Amplification (SISPA) technique. Single strand cDNA was obtained incubating 40 pmol of Primer A (5′-GTTTCCCACTGGAGGATA-N9-3′) and SuperScript III RT Mix (2 μL 5x First-Strand Buffer, 1 μL H_2_O, 1 μL 12.5 mM dNTP mix, 0.5 μL 0.1 M DTT, 0.5 μL SS III RT) (ThermoFisher Scientific, Milan, Italy) at 42 °C for 60 min Second-strand DNA was synthetized by a two-step addiction of Sequenase DNA polymerase (ThermoFisher Scientific), as follows: 5 μL of Mix #1 (1 μL 5x Sequenase Buffer, 3.85 μL H_2_O, 0.15 μL Sequenase enzyme) was incubated 8 min at 37 °C, and then 0.6 μL of Sequenase Mix #2 (0.45 μL Buffer, 0.15 μL Sequenase) was added for further 8 min to complete the second strand synthesis. Primer A-labeled cDNA was then used for the Round B PCR reaction (94 °C for 30 s, 50 °C for 45 s, 72 °C for 60 s, 40 cycles) while using AmpliTaq Gold polymerase (ThermoFisher Scientific) in the presence of 100 pmol Primer B (5′-GTTTCCCACTGGAGGATA-3′).

The PCR products were purified using 1.8x ratio AMPure XP beads (Beckman Coulter, Milan, Italy) in order to maximize the recovery of fragments above 200 bp in size and then eluted in 30 μL of DNase/RNase-free water. The purified products were quantified using the Qubit®DNA High sensitivity Assay Kit (ThermoFisher Scientific). The Nextera DNA Flex Library Preparation kit (llumina, Inc., San Diego, CA, USA) was used to generate multiplexed paired-end sequencing library. Deep sequencing was performed on a MiniSeq 500 platform (Illumina) while using MiniSeq Mid Output Reagent 300 cycles kit to obtain 150 × 2 bp paired-end reads. The raw data were checked for quality using FastQC (https://www.bioinformatics.babraham.ac.uk) and for species confirmation using Kraken2 with MiniKraken2 Database [32]. Subsequently, we processed raw reads by trimming (Trimmomatic ver. 0.38), which led to the removal of any adaptor sequences, leading bases with PHRED < 25 and of trailing bases with PHRED < 25, clipping of the remainder of the read when a sliding window of 20 bases has average PHRED < 25 (Q score > 25) and the removal of reads with length < 36 bases [33].

The mapping of filtered paired-end reads was performed with Geneious^®^ software (version 11.1.5) (Biomatters Ltd, New Zealand) while using Bowtie2 in sensitive-local mode [34] on the *Human metapneumovirus* isolate 00-1 complete genome (Genbank Ref. AF371337.2) to obtain a final consensus. The variant calling was carried out by the Variant Finder Tool (Geneious^®^) filtering out variants with a *p*-value greater than 0, using a minimum variant frequency of 0 and default parameters for Maximum Variant *p*-value (10^−6^) and Minimum Standard-bias *p*-value (10^−5^ when exceeding 65% bias). The minimum sequencing coverage for each variant position was 10 reads. Variant’s frequencies were evaluated as a sum of variant frequencies at that position. Variant analysis was repeated on sequences that were discarded from Bowtie2 alignment setting the minimum overlap identity (50%) and maximum mismatching per read (50%) to manually check the presence of missed HMPV sequences, especially in the region that was suggested by van den Hoogen et al. [35]. Data are available at EBI under study accession n. PRJEB38151.

### 2.10. Statistical Analysis

The data were analysed for significance using non-parametric Wilcoxon signed-rank test. When appropriate, a Student *t* test was applied. *p* < 0.05 was considered to be statistically significant. Statistical analyses were performed using Prism 5 software (GraphPad software, La Jolla, CA, USA).

## 3. Results

### 3.1. HMPV Successfully Infects Human Lung Microvascular Endothelial Cells

**Figure 1:**
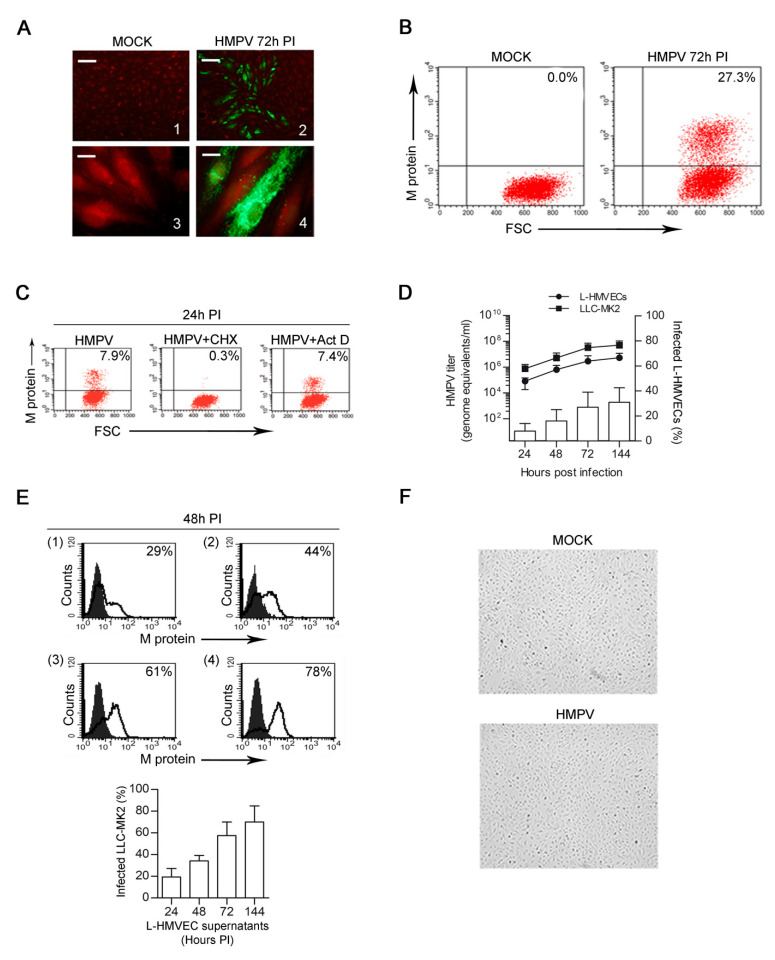
HMPV infection of L-HMVECs. (**A**) Fluorescent microscopy analysis of mock- (left panels) and HMPV-infected (right panels) showing the expression of viral antigens in infected cells. Staining of fixed cells was performed with a mAb to the matrix (M) protein followed by a secondary step with an Alexa Fluor 488 anti-mouse and finally counterstained with Evan’s Blue. Photographs illustrate mock-(1) and HMPV-infected (2) L-HMVECs where a focus of infection is shown (Scale Bar = 50 μm). Enlarged images of mock-(3) or HMPV-infected (4) cells allow to visualize the typical HMPV punctate cytoplasmic staining (Scale Bar = 5 μm). Images are representative of 5 independent experiments. Images were taken 72 h PI (**B**) HMPV-infected L-HMVECs were quantified by flow cytometry. Cells were mock-infected (left panel) or infected (right panel) with infectious HMPV and the presence of the M protein was detected as in (A). Fluorescence was measured using a FACScan and data obtained were analysed using the CELLQuest software. Results are displayed as dot plots. Panel images were taken 72 h PI. Percentages of HMPV^+^ cells are shown in the upper right corner. (**C**) To confirm specificity of M protein staining, L-HMVECs were infected with HMPV in the presence or absence of 30 μM cycloheximide or 1 μM actinomycin D. Cells were analysed at 24 h PI (**D**) HMPV replication kinetic in L-HMVECs. Left scale: L-HMVECs were infected and cell culture supernatant samples were collected at the indicated time points. Quantitation over time of HMPV RNA present in purified nucleic acid derived from infected L-HMVEC (●) was determined by rtPCR. RNA derived from the permissive cell line LLC-MK2 infected with HMPV served as control of replication (■). Data indicate mean viral loads (number of copies per mL of supernatant) ± SD of five independent experiments. Each quantitation was performed in three replicates. SD of the triplicate never exceeded 0.5%. Right scale: % of HMPV-infected L-HMVECs was quantified by flow cytometry, as described in A. Bars represent mean ± SD of five independent experiments (**E**) Release of infectious particles by L-HMVECs. LLC-MK2 were exposed for 3 h to the supernatants of HMPV-infected L-HMVECs harvested 24 (1), 48 (2), 72 (3), and 144 (4) h PI. After two days, the infected LLC-MK2 cells were quantified by flow cytometry. Fluorescence was measured using a FACScan and data obtained were analysed while using the CELLQuest software. In the upper panel histograms from one representative experiment out of three with similar results are shown. Solid graphs represent data obtained from LLC-MK2 exposed to supernatants derived from mock L-HMVECs. Percentages of HMPV^+^ cells are shown in the upper right corner of each panel. In the lower panel, bars represent the mean ± SD of the % of HMPV-infected cells detected in all the experiments performed (**F**) Light microscopy evaluation of uninfected (upper panel) and HMPV-infected monolayers (lower panel) at 6 days PI. ECs completely retain their characteristic morphology (Scale Bar = 50 μm).

Because of lung anatomy, during HMPV acute infection, pulmonary microvessels (ECs) are highly exposed to virus contact. We first examined the ability of HMPV to replicate in L-HMVECs in order to assess if microvascular ECs can represent a relevant target for HMPV infection. Immunofluorescence analysis showed that L-HMVEC cultures consistently allowed HMPV replication within 72 h PI. The foci of cells presenting specific granular cytoplasmatic staining typical of HMPV could be observed in HMPV-infected cells, whereas fluorescence was always absent in uninfected cells, as shown in Figure 1A. At that time, more than 25% of HMPV-infected L-HMVECs displayed the presence of HMPV M antigen (Figure 1B), as determined by flow cytometry. The ability of virus to replicate in L-HMVECs was next confirmed by infecting them in the presence of cycloheximide. After virus adsorption, cells were washed and fresh medium containing the protein synthesis inhibitor was added. The inhibition of *de novo* protein synthesis with cycloheximide, via its ability to block the peptidyl synthetase activity of eukaryotic ribosomes, leads to the lack of viral protein synthesis and, consequently, no more positive cells were observed. As expected, the addition of actinomycin D, which inhibits DNA-dependent RNA synthesis specifically without affecting the viral RNA-dependent RNA synthesis, did not abolish the production of viral proteins (Figure 1C).

The cells were infected, cultured for 24, 48, 72, and 144 h PI, and the supernatant tested at each time point for viral load in order to evaluate whether HMPV infection of L-HMVECs resulted in the release of virus particles. The quantitation of viral RNA by rtPCR showed the presence of HMPV genome/equivalents at all of the investigated time points. A constant increase of extracellular viral RNA amount was observed, presumably from progeny virions that were released in the cell supernatants by HMPV-infected L-HMVECs. The rate of viral titer increases in L-HMVECs paralleled the one that was observed in the supernatants of the permissive cell line LLC-MK2 over the same time period. At the same time points, an increase in the percentage (%) of infected L-HMVECs was also observed (Figure 1D). HMPV-infected L-HMVECs released infectious virus particles, as determined by infectivity studies with LLC-MK2 cells. Supernatants that were obtained from HMPV-infected L-HMVECs (HMPV-secretome) harvested at different time PI were all capable of reinfecting LLC-MK2 cells. The amount of infectious virus present in L-HMVECs supernatants increases during time, as determined by the growing % of infected LLC-MK2 cells detected 48 h after their infection with HMPV-secretome (Figure 1E), and strictly correlate with the RNA viral load that was quantified by rtPCR. Interestingly, the infected L-HMVECs exhibited a morphology similar to that of mock cultures, as no cytopathic effect was observed (Figure 1F). Taken together, these data indicate that HMPV is capable of sustaining a productive infection in L-HMVECs.

### 3.2. HMPV Establishes a Persistent Productive Infection in L-HMVECs

**Figure 2:**
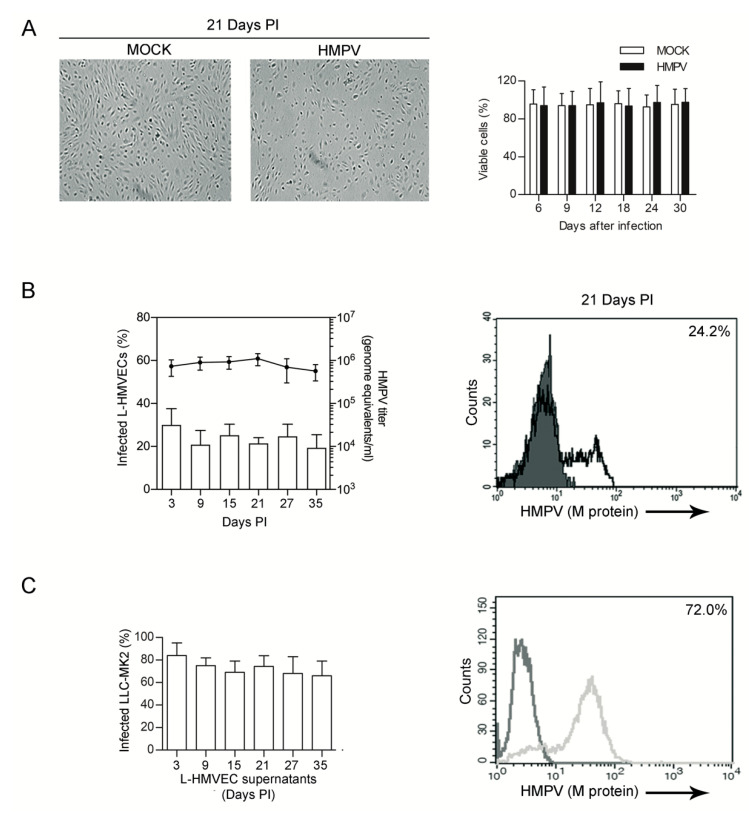
HMPV persistent infection of L-HMVECs (**A**) Left panel: Light microscopy evaluation of mock and HMPV-infected monolayers. At the third week of infection (day 21 PI), infected cells appear less abundant, but retain the EC characteristic morphology (Scale Bar = 50 μm). Right panel: Bars represent % of mock (□) and HMPV-infected (■) viable cells at the indicated time points, as assessed by trypan blue exclusion assay. (**B**) Release of virus by long term cultured L-HMVECs. Left panel: Bars (left axis scale) indicate the % of HMPV-infected L-HMVECs, as assessed by flow cytometry, at the indicated time of collection. Line (right axis scale) are mean viral loads (number of genome copies per mL of supernatant) at the indicated time of collection. The viral titer in culture supernatant was always determined 72 h after the culture medium was changed. Data are means ± SD from 3 independent experiments. Right panel: Representative flow cytometric analysis of infected L-HMVECs at 21 days PI. HMPV M antigen expression was evaluated as described in Figure 1. Percentage of HMPV^+^ cells is shown in the panel upper right corner. (**C**) Release of infectious particles by persistently infected L-HMVECs. LLC-MK2 were exposed for 3 h to the supernatants of HMPV-infected L-HMVECs collected at different time points. After six days, the rate of HMPV-infected LLC-MK2 cells was quantified by flow cytometry. Left panel: Bars represent the mean ± SD of the % of infected LLC-MK2 detected in 3 independent experiments. In the horizontal axis are indicated the day of harvesting of HMPV-infected supernatants from L-HMVECs. In the right panel a representative histogram of LLC-MK2 infected with supernatants from HMPV-infected L-HMVECs at 21 days PI is shown. Light histogram represents data obtained from LLC-MK2 exposed to supernatants derived from mock L-HMVECs.

ECs are permissive to infections with DNA and RNA viruses and, in many cases, they are capable of sustaining a persistent infection in the absence of obvious cytopathic effects. In vivo, HMPV can establish a persistent infection both in humans [36] and in animal models [19,20], but, so far, no data are available regarding its ability to persist in ECs. Therefore, we sought to verify whether L-HMVECs can support a productive HMPV infection for an extended period of time. Mock and HMPV-infected L-HMVECs were cultured for > 30 days with no significant loss of viability, even if, starting at the third week of infection, the growth of infected L-HMVECs slowed down and cells failed to reach the complete confluence (Figure 2A). Independently from this event, L-HMVECs continued to release virus during their lifespan in vitro: after the increase in viral titer observed during the first six days of culture, HMPV copies detected in the cell supernatant remained stable throughout the subsequent period of observation, despite cell subculturing being necessary for maintaining viable cultures. Accordingly, a fair amount of L-HMVECs supports a detectable viral replication, as assessed by the expression of viral proteins within cells and the presence of viral RNA in the cell supernatants at each time point of analysis (Figure 2B). L-HMVECs released infectious virions, as in the acute phase of infection. In fact, supernatants from L-HMVECs harvested at different times PI were all capable of productively infecting LLC-MK2 (Figure 2C). This evidence suggests that ECs can contribute to HMPV release, not only during the acute phase of infection, but in the event of persistence in the respiratory system.

### 3.3. HMPV-Infected L-HMVEC Secretomes Induce DC to Promote a Th2 Phenotype

**Figure 3:**
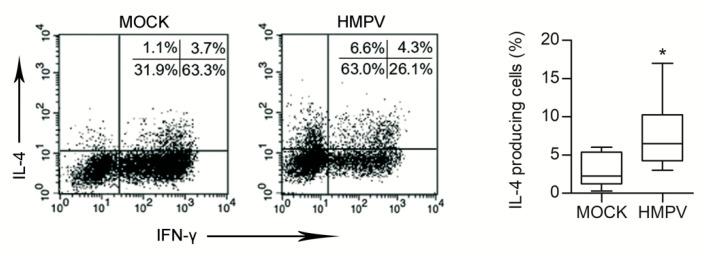
DCs primed by secretomes of HMPV-infected L-HMVECs induce Th2 polarized cells. Purified CD4^+^ T cells were co-cultured with allogenic DCs pretreated with mock or HMPV-infected L-HMVEC secretomes harvested at 21 days PI. After 7 days cells were treated with PMA, Ionomycin, and Brefeldine, then fixed, permeabilized, stained for IFN-γ and IL-4 and analysed in flow cytometry as described in Section 2. Cell gating was on CD4^+^ T cell population. In the left panel intracellular cytokine expression is represented as dot-plots. Numbers indicate the % of positive cells in each quadrant. Data represent one of 10 independent experiments performed with cells from different donors. Box-plot graph (right panel) represents % of IL-4-producing cells generated after the allogenic culture of naïve CD4 T cells with DCs treated with mock or HMPV-infected L-HMVEC secretomes. Lower quartile, median, upper quartile, and largest observations are represented. * *p* < 0.05.

During viral infections, airway immDCs act as sentinels that are capable of initiating pulmonary immunity [37]. By bridging innate to adaptive immune response, lung DCs can favour the development of different T helper [38] phenotypes. DC effector functions heavily depend on the microenvironmental context, as they are influenced by a crosstalk with neighbouring cells [39,40]. Clinical reports suggest that HMPV infection during infancy might be a significant risk factor for asthma development [10] or exacerbation [41]. Moreover, experimental data indicate that adaptive immune response during the late stages of HMPV infection skews toward a Th2 response [8,42]. For these reasons, we evaluated whether secretome derived from HMPV-infected lung ECs could prime DCs toward an inappropriate Th2 T cell immunity. ImmDCs, generated from monocytes, were treated for 48 h with secretomes that were derived from mock and HMPV-infected L-HMVEC cultures collected at 21 days PI, then cocultured for seven days with allogenic naïve CD4 T lymphocytes. DCs ability to promote naïve T cell differentiation toward a Th2 phenotype was then assessed by evaluating the amount of IL-4-producing T cells generated in the culture. Flow cytometric analysis revealed that, when DCs were pretreated with secretome obtained from HMPV-infected L-HMVECs, a higher % of IL-4^+^ T cells were generated in comparison to the ones obtained by culturing T cells with DC treated with secretome from mock L-HMVECs (Figure 3). Of note, in parallel to IL-4 increase, a reduced frequency of IFN-γ-producing CD4^+^ T cells was generated in the coculture that was primed by HMPV secretome-treated DCs.

### 3.4. HMPV-Infected LHMVECs Secretome Induces OX40L Expression on DCs

Upregulation of OX40L expression on the DCs surface is known to trigger a Th2 response [43]. We therefore sought to clarify if the observed pro-Th2 activity of DCs treated with HMPV-infected secretome was dependent from OX40L expression.

**Figure 4:**
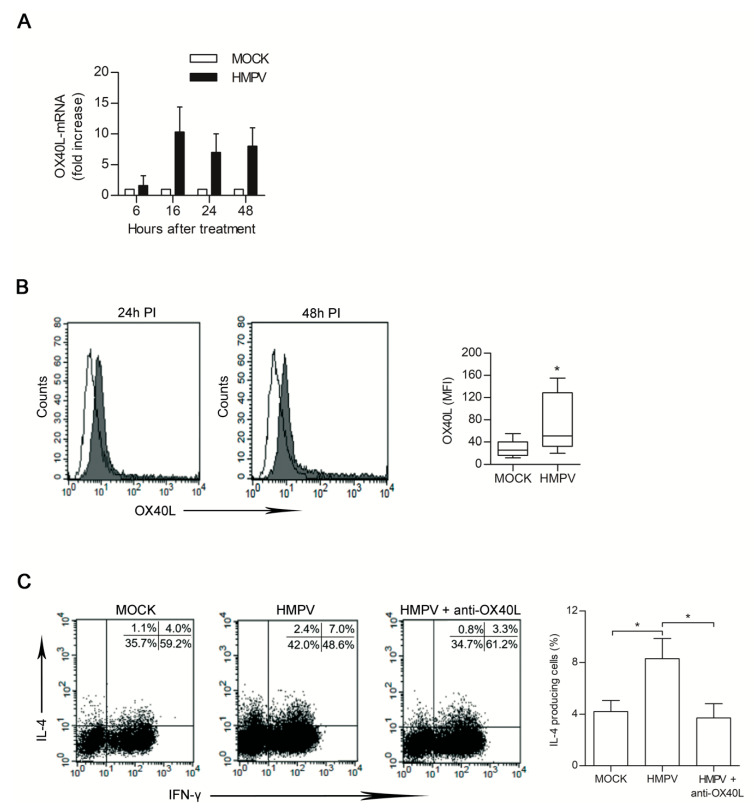
HMPV-infected secretome (21 days PI) induces OX40L expression in dendritic cells (DCs). (**A**) DCs treated with mock and HMPV-secretome were harvested at the indicated times. OX40L transcripts were analysed by rtPCR and mRNA levels were normalized using β-actin mRNAs detected in the same samples. Results are expressed as fold induction over the levels detected in mock cells at the same time points. Bars represent the mean ± SD of five independent experiments. (**B**) Left panel: Flow cytometric analysis of OX40L expression on DC surface. DCs were analysed for OX40L expression upon 24 h and 48 h of treatment with mock (open line) or HMPV-infected (solid line) secretomes. Histograms are from one representative experiment. Box-plots in the right panel show the mean florescence intensity (MFI) values of OX40L expression from 10 independent experiments. Lower quartile, median, upper quartile, and largest observations are represented. * *p* < 0.05. (**C**) Naïve CD4 T cells were cultured with DCs primed with HMPV-infected secretome in the presence or absence of anti-OX40L neutralizing mAb. After seven days, cells were analysed by flow cytometry for intracellular expression of IFN-γ and IL-4. Dot-plots in the left panel represent one out of the three independent experiments performed; numbers indicate the % of positive cells in each quadrant. Bars in the right panel represent the mean % ± SEM of IL-4-producing cells of three performed experiments upon the indicated treatment. * *p* < 0.05.

DCs were treated with HMPV and mock secretome and analysed at different time post-treatment for the up-regulation of OX40L specific mRNAs. Time course-experiments showed that increase of OX40L gene expression was not observed within few hours of treatment but became evident starting from 16 h post-treatment (Figure 4A). The level of OX40L protein on DC surface was then evaluated by flow cytometry. In comparison to the amount of protein expressed by DC treated with mock secretome, an increase in OX40L expression was observed when DCs were treated for 24 h or 48 h with HMPV-secretome (Figure 4B). To prove that OX40L was involved in the Th2 polarization evidenced in our experimental system, neutralization experiments were performed. DCs pretreated with HMPV-secretome were cocultured with naïve T cells in the presence of neutralizing antibody specific for OX40L. Upon this condition, the % of IL-4-producing T cells generated after 7 days of DC/T cell coculture significantly decreased, returning to the level observed when DCs were treated with mock secretome (Figure 4C).

### 3.5. Th2 Polarization is Directly Induced by HMPV Viral Particles Contained in L-HMVEC Secretomes

**Figure 5:**
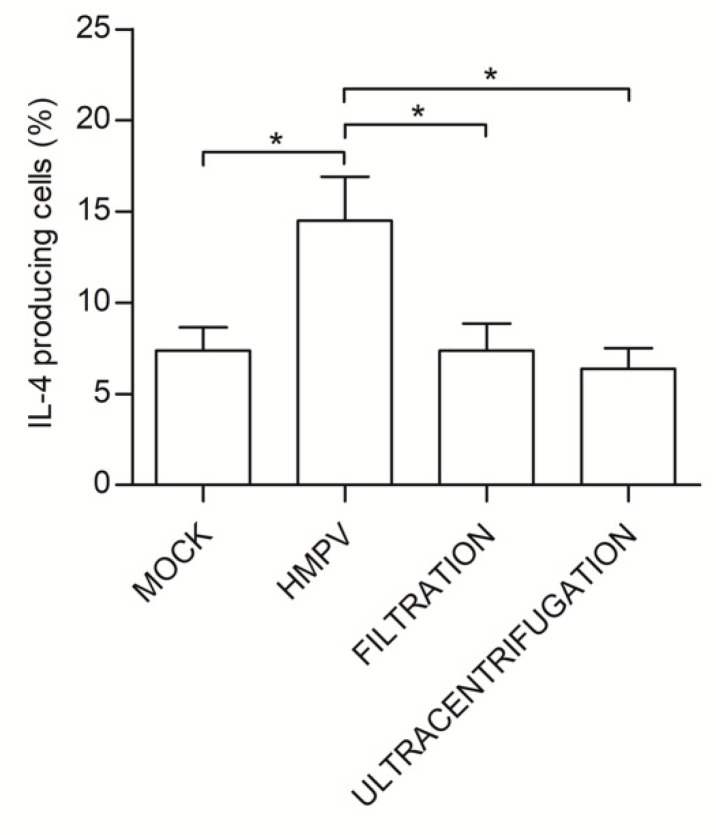
Virus clearance from HMPV-infected secretome abolishes the pro-Th2 effects. The DCs were treated for 48 h with mock secretome, HMPV-infected secretome or with secretomes derived from HMPV-infected L-HMVECs where the virus was cleared by size exclusion (filtration) or ultracentrifugation. DCs were then co-incubated with allogenic naïve CD4 T cells for seven days. Bars represent the mean ± SEM of IL-4-producing cells (%) detected upon the described culture conditions. The results are from four independent experiments. * *p* < 0.05.

TSLP is known to be released by epithelial cells upon acute HMPV infection, contributing to the shaping of initial anti-HMPV immune response [17]. We removed viral particles by Amicon (Millipore, Milan, Italy) filtration (size cut-off 100,000 Dalton) in order to understand if the Th2 polarization observed when naïve T cells were treated with L-HMVEC secretomes was dependent on TSLP or other soluble factors produced by the infected ECs. The lack of infectivity of these supernatants was verified by their inability to reinfect LLC-MK2. No viral antigens were observed upon a six days long follow up of treated LLC-MK2 by flow cytometry and, accordingly, no increase in genome equivalents was detected. Unexpectedly, when immDCs were stimulated for 48 h with HMPV-clarified secretomes, the % of IL-4-producing T cells generated from the DC/naïve T cells coculture were superimposable to that obtained with secretome derived from mock L-HMVECs. In support of this result, no TSLP protein was detected in L-HMVEC secretomes that were harvested in both the acute and persistent phase of infection. HMPV removal was also performed also by ultracentrifugation to verify that 100,000 cut-off filtration did not eliminate some other critical, yet unknown, soluble factors of high molecular weight. Again, the procedure was efficient in eliminating secretome infectivity and results obtained using this HMPV-free secretome confirm that the presence of virus particles in secretome plays a key role in inducing DCs toward a pro-Th2 effector phenotype (Figure 5).

### 3.6. HMPV Induces, per se, Th2 cell Polarization

**Figure 6:**
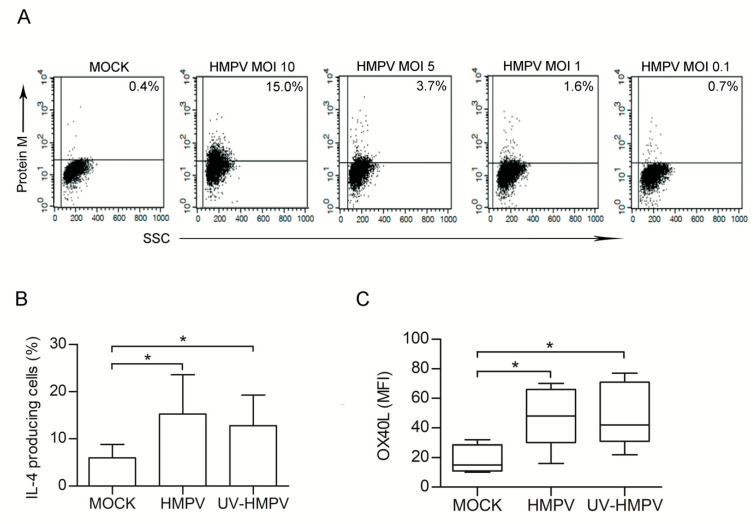
HMPV is capable, *per se*, to skew DCs toward a pro-Th2 phenotype. (**A**) DCs were left untreated or infected with HMPV at different MOIs (10, 5, 1, 0.1). After 48 h cells analysed by flow cytometry for the intracellular expression of the viral M protein. Dot plots show the fluorescence values vs the side scatter. Numbers in the upper right corner represent the % of cells expressing HMPV. Results are from one representative experiment out of 4 performed. (**B**) DCs were left untreated (MOCK) or treated for 48 h with UV-inactivated (UV-HMPV) or infectious (HMPV) HMPV at MOI 1. Allogenic coculture of DCs with naïve T cells was then performed and infectious IL-4 expression was evaluated as in Figure 3. Bars represent the mean ± SEM of IL-4-producing cells (%) detected upon the described culture conditions. Results are from one representative experiment out of four performed. * *p* < 0.05 (**C**) OX40L expression on DCs surface was evaluated upon 48 h of treatment with medium (MOCK) UV-inactivated (UV-HMPV) or infectious (HMPV) virus at MOI 1. Box-plots show the MFI values of OX40L expression detected in eight independent experiments. Lower quartile, median, upper quartile, and largest observations are represented. * *p* < 0.05.

Next, we investigated whether HMPV could, *per se*, up-regulate OX40L expression and skews DC toward a pro-Th2 phenotype. To this purpose, we performed experiments using purified HMPV particles. First, we aimed to evaluate whether a productive infection of DCs occurred. ImmDCs were treated with HMPV at different MOIs (10, 5, 1, 0.1), cultured for 48 h, and then intracellular flow cytometry analysis was performed to establish the % of infected cells. In accordance with the data obtained by other Authors [44], we observed that DCs have a low susceptibility to HMPV infection. No viral protein could be detected upon infection with HMPV at MOI 0.1. A low % of HMPV-infected DCs was observed at higher MOI (median %: 2.5% at MOI 5 and 1.5% at MOI 1). At MOI 10, despite a higher % of cells being HMPV^+^ (from 4 to 15%), we observed a high mortality rate. None of these cells released virions within 48 h of infection (Figure 6A).

DCs were infected with HMPV at MOI 1, and then co-cultured with allogenic naïve CD4 T lymphocytes to verify whether the pro-Th2 effect observed when DCs were treated with HMPV-secretome could be reproduced by a comparable amount of purified HMPV particles. After seven days % of IL-4^+^ cells were analysed. Cocolture of naïve CD4 T cells with HMPV-treated DCs induced a significant increase of Th2 cells, as compared to the untreated counterpart (Figure 6B). Moreover, HMPV infection of DCs at MOI 1 induced the expression of OX40L (Figure 6C). Finally, UV-inactivated HMPV preparation was used in order to confirm that cell polarization mediated by HMPV did not depend on viral replication in DCs. Non-replicating HMPV still induced Th2 polarization and increased the surface expression of OX40L, as shown in Figure 6B,C. In conclusion, the presence of HMPV particles in the culture was sufficient to prime DCs toward a pro-Th2 phenotype through up-regulation of OX40L expression.

### 3.7. Full Genome Sequencing of HMPV

**Figure 7:**
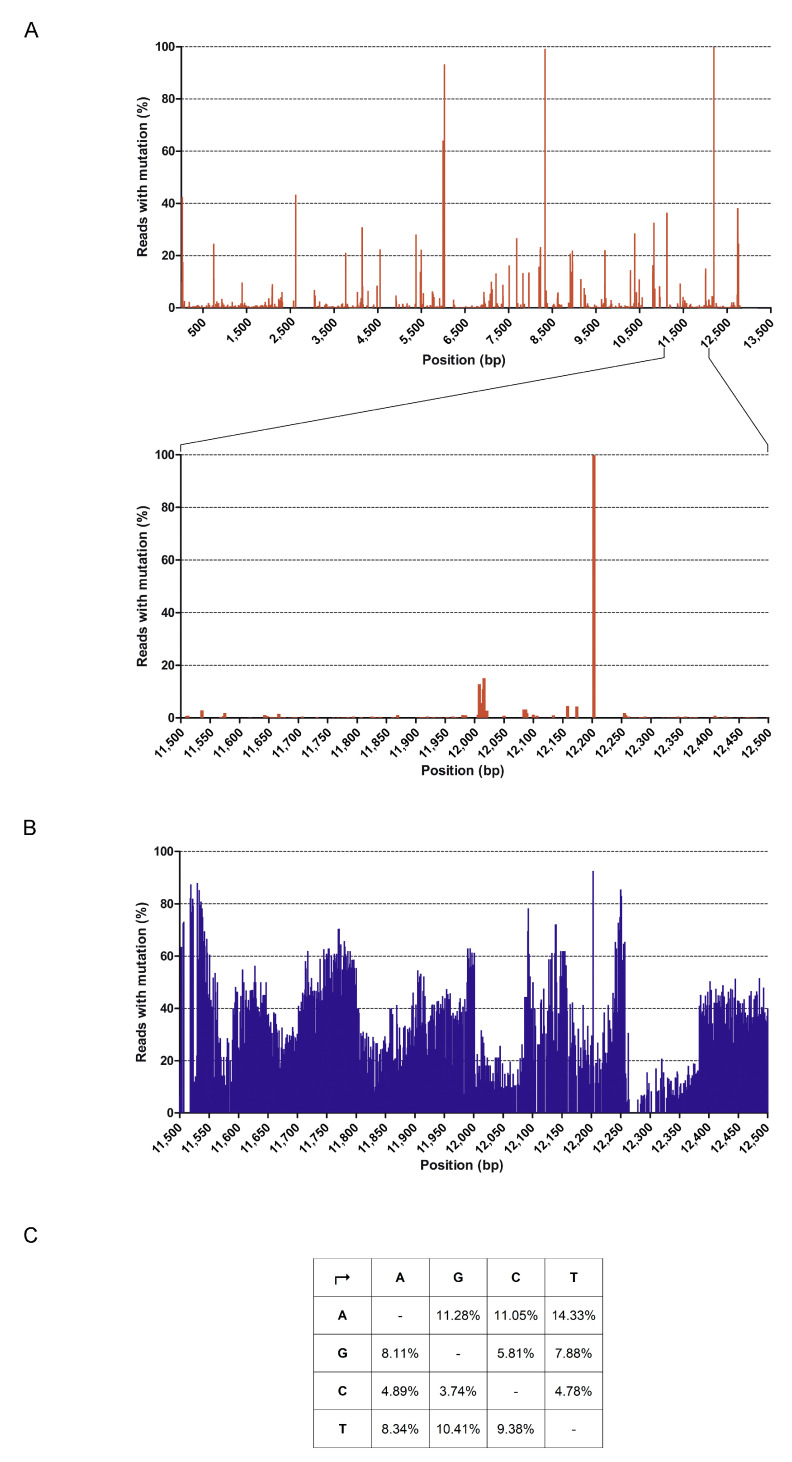
Sequence variability detected in the HMPV stock. (**A**) The upper graph represents the % of reads displaying mutations in comparison to the reference sequence AF371337.2 at each nt position detected, as assessed by NGS. Nt positions in the HMPV genome are reported in the *x* axis. An enlargement of nt positions 11,500–12,500 is also shown (lower graph). (**B**) Graph shows sequence variability at nt positions 11,500–12,500. Alignment to AF371337.2 was performed using NGS reads obtained by lowering homology parameters. (**C**) Table represents the % of bases substitution occurring at each nt position in comparison to Ref AF371337.2. Analysis was performed for the genome region at nt 11,500–12,500.

HMPV stock was subjected to sequence independent Single-Primer Amplification (SISPA) technique, in combination with Illumina NGS, to better characterize the viral population capable of determining the pro-Th2 effect observed. This method has allowed obtaining a non-selective amplification of HMPV sequences that were present in the viral stock, thus permitting to characterize viral genetic variability. Trimmed reads were mapped on “*Human metapneumovirus* isolate 00-1 complete genome” (Genbank Ref. AF371337.2) showing coverage of 99.4% supported by 1192,417 mapped reads, as assessed by local alignment. Analysis of the HMPV consensus sequence has revealed that, although a low level of variability is detectable in virtually any position of the viral genome, only few SNPs were detectable (Figure 7A). Of note, five of the seven SNPs detected in > 40% of the total reads corresponded to silent substitutions, whereas the other two SNPs in position 5999 (Alanine to Valine) and 8035 (Tryptophan to Leucin) were substitutions that were already identified in other HMPV isolates.

For the identification of possible divergent variants of HMPV, a more detailed analysis was performed by also evaluating the sequences discarded using the standard alignment criteria. Setting parameters of overlap identity and mismatching per reads to 50%, we recovered other 84,231 putative HMPV sequences that only partially aligned with the reference and mapped throughout the genome (Figure 7B). Within these sequences, we manually checked for the presence of the hypermutated, excessively edited sequences that were described by other Authors [35]. In their paper, van den Hoogen et al. demonstrated the presence of defective viral particles with polymerase gene hypermutated starting beyond nucleotide (nt) 11,000. Hypermutation was due to extensive editing, generating high levels of A to G (53%) and T to C (36%) substitutions. We performed a single base variant calling in the region of nt 11,500–12,500 in order to understand if HMPV sequences enriched in specific A to G and T to C substitutions were present in the viral stock. No appreciable differences in substitution rates occurred and substitutions were equally distributed among all the possible combinations, as shown in Figure 7C. The obtained results were confirmed by a de novo assembly and subsequent mapping of the contigs on the AF371337.2 reference.

Taken together, these results indicate that the HMPV stock used in this study is not significantly different from the originally described 00-1 strain.

## 4. Discussion

Persistence in the host and impairment of T cell activation are critical mechanisms that are exploited by HMPV to avoid virus clearance [14]. However, viral strategies that are used to circumvent the host immune system are still poorly understood.

In this study, we have demonstrated that primary L-HMVECs support a productive in vitro infection of HMPV, showing no evidence of a cytopathic effect over the first month PI and, interestingly, this persistent state of infection was characterized by a continuous release of viral particles. Productive infection in the absence of cytopathic effect is an essential prerequisite for the establishment of viral persistence. Our data show that levels of HMPV compatible with the viral load present in the course of a natural infection are sufficient to infect a relevant number of lung ECs and that an appreciable and constant % of L-HMVECs sustains viral replication. The presence of cellular subsets acting as reservoirs represents the basis for viral persistence. We have recently described that, in alveolar epithelial cells A549, HMPV infection leads to massive cytopathic effects, but a small subset of cells can acquire a reservoir cell phenotype with a long-lasting low-level production of infectious virus [21]. Our in vitro results are in agreement with recent findings described for Polyoma BK in the renal microenvironments, where the Authors hypothesized that viral persistence is mostly maintained in ECs, while productive infections linked to pathology mainly occur in epithelial cells [26]. Therefore, we can hypothesize that, similarly to other viruses [24,25,26], HMPV might use lung microvascular endothelium as a privileged site of viral persistence.

It is a matter of facts that, during respiratory infections, epithelial cells actively contribute to the development of the early adaptive immune response, mostly by releasing soluble mediators helpful to prime DC functions. In the case of HMPV infection, human alveolar cells significantly increase the expression of pro-Th2 mediators [17] that could contribute to the inefficient elimination of infected cells within the airways. So far, few hypotheses on the eventual role of other target cells have been posed. Here, we have shown that, once HMPV infection is established and maintained in endothelial reservoir, these cells could act as bystander cells, as soluble factors that are released by HMPV-infected ECs are dispensable for the priming of adaptive immunity. However, relevant to the HMPV-induced immunopathogenesis, lung microvascular cells release viral particles that are capable of influencing DC functions. Several groups have demonstrated that DCs are activated upon their interaction with HMPV, despite their maturation response is modest when compared to the one induced by other viruses [44,45,46,47]. In an experimental mouse model, HMPV has been described as capable of dampening the activation of naïve CD4 T cells [44], data that are different from those obtained in human cells where no inhibition of T-cell activation induced by HMPV-inoculated monocytes-derived DCs has been observed [47]. Our results suggest that, rather than inhibiting T cell maturation, the presence of HMPV in the microenvironment equips DCs with the capacity to skew naïve T cell priming toward a Th2 phenotype and that DC-expressed OX40L plays a role in this function. In physiological conditions, HMPV infects DCs in a restrictive and abortive manner, so the release of infectious particles is dispensable for the alteration of their functions [44]. In our experimental system, we observed that a small % of cells shows the presence of viral antigens upon infection, but the same they were capable of priming DC toward a pro-Th2 phenotype and up-regulate OX40L expression. Further, the biological effects that were exerted by HMPV on DC function were dispensable from the ability of virus to replicate within DCs, as demonstrated by the results that were obtained using UV-treated virus. It has been demonstrated that myeloid DCs activated by TSLP can promote the differentiation of naïve CD4 T cells into a Th2 phenotype in a unique manner dependent on OX40L [48]. Moreover, the repression of OX40L by pharmacological treatment with statins suppresses the DC-mediated Th2 response [43]. Therefore, HMPV could act as a TSLP-mimicking agent inducing DCs to upregulate OX40L expression and to favor the generation of Th2-prone cells. In agreement with our findings are the results obtained by Lay et al. [17] in a mouse experimental model of infection lacking TSLPR expression. They observed that OX40L expression does not exclusively depend on TSLPR signaling and suggested that the effector functions of OX40L on the surface of DCs may be highly dependent on the milieu on which they mediate downstream processes. Wheezing from viral infections is associated with an increased likelihood of subsequent recurrent wheezing and asthma [10,49]. Recently, Coverstone et al. [50] investigated the risk for persistent wheezing and asthma later in childhood in children who experienced severe LRTI with HMPV, concluding that, similar to rhinovirus and RSV, HMPV confers an increased risk of recurrent wheezing in children infected early in life. The role of OX40/OX40L interaction in Th2-mediated diseases is well described in animal models: OX40L-deficient mice have an impaired ability to generate Th2 immune responses and develop pulmonary lung inflammation and airway hyperreactivity in a murine model of asthma [51]. Moreover, anti-OX40L antibodies can abrogate Th2-induced pathologies in experimental leishmaniasis [52]. Evidences supporting the role of OX40/OX40L axis in human Th2 diseases are difficult to accumulate and this is because the interaction between OX40/OX40L in Th2 polarization might occur early in the disease pathogenesis and it is primarily located in the lymph node, where the adaptive immune response first occurs [53]. However, OX40L is now considered to be a promising target for the prevention of allergen-induced airway obstruction and clinical trials are ongoing. The results shown here suggest that HMPV induces the up-regulation of OX40L on DCs, which predisposes them to skew the T cell response toward a Th2 phenotype and this might contribute to the development or exacerbation of HMPV-associated chronic airway diseases. Since the Th2 response is mostly seen in the later stages of HMPV infection [42], it is tempting to speculate that HMPV persistence in cellular lung reservoirs helps to sustain these aspects of the viral pathogenesis.

Defective viral genomes have been described in most RNA viruses and they are recognized as danger signals for triggering of antiviral immunity in many infections, thus altering DC functions [54]. The experiments shown in this research have been performed using a viral stock of the HMPV wild strain NL-001. For this virus, it has been demonstrated that passages at high MOI in Vero 118 cells lead to the accumulation of defective viral particles that are characterized by hypermutation of the genome due to active cytoplasmic editing of viral RNAs [54]. Our NGS data show that, despite that a certain degree of random sequence variability arises within the single HMPV inoculum we used, the presence of a dominant type of defective genomes characterized by extreme editing has not been observed. This evidence suggests that the pro-Th2 effect that is induced by HMPV is not dependent on the presence of hypermutated defective viral genomes within the viral preparation.

In conclusion, our study contributes to better understanding how HMPV prompts the development of atypical T cell immunity and might be helpful in identifying new targets for the prevention of HMPV-associated long-term consequence.
